# Supplementary material for: A general fluorescent light-up probe for staining and quantifying protein
Source: R Soc Open Sci. 2019 Aug 28;6(8):190580. doi: 10.1098/rsos.190580 (PMC6731743; doi:10.1098/rsos.190580)
Supplement: Electronic Supplementary Material for A General Fluorescent Light-Up Probe for Staining and Quantifying Protein [file rsos190580supp1.docx]

Electronic Supplementary Material

A General Fluorescent Light-Up Probe for Staining and Quantifying Protein

Jiawei Zou^ab^, Gangyi Chen^a^, Feng Du^a^, Yi Yuan^a^, Xin Huang^a^, Juan Dong^a^, Kexin Xie^a^, Xin Cui^a^, Zhuo Tang*^a^

a Natural Products Research Center, Chengdu Institution of Biology, Chinese Academy of Science, Chengdu 610041, P. R. China

b University of Chinese Academy of Sciences, Beijing,100049, P.R.China

*Correspondence: [tangzhuo@cib.ac.cn](mailto:tangzhuo@cib.ac.cn)

**Table of content**

1. **Experimental Section2-3**
2. **Fluorescence images of E. coli cells stained with PyMDI-Zn4**

Figure S1

1. **Comparison experiment on “with or without washing” in gel** **5**

Figure S2

1. **Interference experiments in the presence of non-protein substances6**

Table S1

Figure S3

1. **Comparison experiment** **on SYPRO Orange and PyMDI-Zn7**

Figure S4

1. **Cellular location with PyMDI-Zn8-9**

Figure S5

Figure S6

1. **Cell viability assays** **10**

Figure S7

1. **Protein quantitation 11**

Figure S8

1. **Notes and references12**
2. Experimental Section
   1. Materials

Bovine serum albumin (BSA), Human serum albumin (HSA), Brilliant blue R–250, PyroninY, DAPI and SYPRO (R) Orange Protein Gel Stain were purchased from Sigma Chemicals Co. All other chemicals are analytical reagent grade. JM109, DH10B, BL21(DE3) Chemically Competent Cell, ProteinRuler I and ProteinRuler II were purchased from TransGen Biotech (Beijing, China). Pierce Rapid Gold BCA Protein Assay Kit was purchased from Thermo Fisher Scientific. RecR, RuvA, RuvB, RecA and MutL were cloned into an expression vector, and the proteins were purified. Cell cultures were maintained in DMEM medium supplemented with 10% heat-inactivated fetal calf serum, 100 u/ml penicillin and 100 μg/ml streptomycin, at 37 °C in a humidified atmosphere containing 5% CO2. All the solutions were prepared with sterile ultrapure water. The (Z)-1,2-dimethyl- 4-(pyridin-2-ylmethylene)-1H-imidazol-5(4H)-one (PyMDI) was synthesized according to the synthesis procedure described in Ref.^1^.

- 1. Instruments

Absorbance, excitation and emission spectra were measured with a Thermo Scientific Varioskan Flash. Flow cytometry experiments were performed on MoFlo^TM^ XDP Cell Sorter (Beckman Coulter). Cells images were recorded using a Leica TCS SP8 Confocal Microscope. Laser 355 nm and 488 nm were selected when using Flow cytometer, and laser 405 nm, 488 nm and 552nm for Confocal Microscope. SDS-PAGE was performed on the Invitrogen SureCast^TM^ system, and the gel images were recorded by GE Healthcare Bio-Sciences AB Typhoon FLA 7000 and Canon Digital camera EOS 600D. Kjeldahl method was performed on Foss Kjeltec 2200.

- 1. Electrophoresis and Staining with PyMDI

Proteins were loaded in 12% SDS-polyacrylamide gel. The stacking layer runs at 40 v, and the resolving layer runs at 80 v. The gel was rinsed with deionized water, and stained with PyMDI-Zn (100 μM) for 5 minutes at room temperature (25 ℃). Coomassie stain protocol: the SDS-PAGE gel was rinsed with deionized water, and then stained with coomassie solution, incubation with gentle agitation at room temperature for 1-20 hours. Gels were destained overnight with gentle agitation.

- 1. Cellular location

Hela, and HepG2 cells grown in 20 mm glass bottom cell culture dish, cells were washed with PBS for 3 times, followed by incubation with PyMDI-Zn (50 μM), DAPI (nucleus location because of binding to DNAs) and PyroninY (nucleolus location because of binding RNAs) for 30 minutes at 37 ℃. And samples were viewed under a Leica TCS SP8 Confocal Microscope. Images were obtained using the LAS AF software, then subsequently processed with the Adobe Photoshop program.

- 1. Cell viability assay

HepG2 was cultured in a 96-well plate, the cells were exposed to PyMDI-Zn at a concentration in the range of 50~500 μМ (DMSO as a control) for 24 hours, then the medium was replaced with 200 μl DMEM 10%(v/v) Alamar Blue. After 2 or 3 hours, fluorescence was monitored at wavelength 530 nm for excitation and 590 nm for emission by Thermo Scientific Varioskan Flash.

- 1. Determination of sample protein concentration

100 μl protein solution containing different amounts of protein (20 to 600 μg) with 100 μM PyMDI-Zn were incubated at room temperature for about 5 minutes, and 100 μl ddH2O with 100 μM PyMDI-Zn was used as background. Then the fluorescence of those samples was detected at 520nm with excitation at 486 nm by Thermo Scientific Varioskan Flash. The weight of protein was plotted against the corresponding fluorescent intensity to obtain a standard curve. The protein concentration of the unknown samples was converted by the linear equation from the standard curve. Protein solutions are normally assayed in triplicate.

1. Fluorescence images of E. coli cells stained with PyMDI-Zn


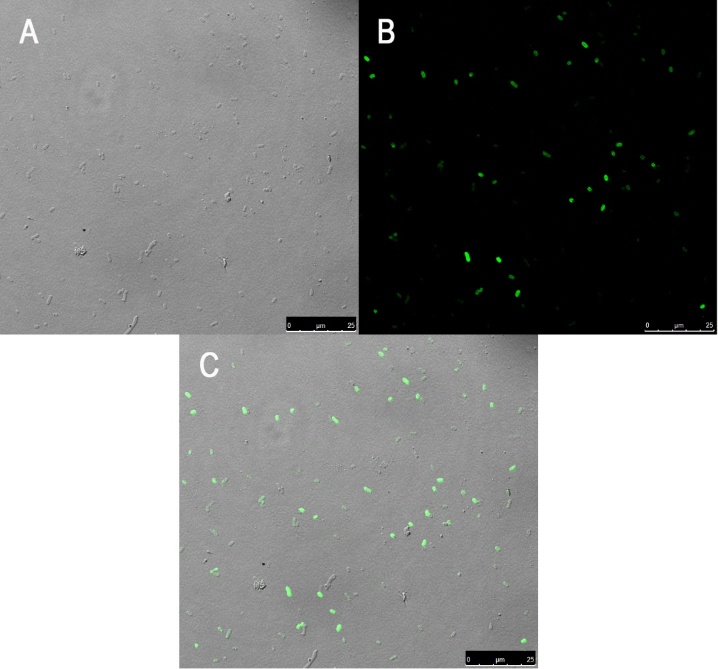


**Figure S1** Fluorescence images of E. coli cells after incubation with PyMDI-Zn. Inoculate 5 ml of LB medium with 50 μl of overnight culture, then incubate the cultures for about 2 hours at 37 ℃ until cells reach mid-log growth. E. coli cells were washed with deionized water for 3 times, followed by incubation with PyMDI-Zn (50 μM), for 30 minutes at 37 ℃. Samples were dropped on the slide, then performed under Leica TCS SP8 Confocal Microscope. (A) bright-field image (B) E. coli cells were stained with PyMDI-Zn, and excited at 488 nm. E. coli were basically green-stained. (C) the merged image of bright-field and PyMDI-Zn. Scale bars 25 μm

1. Comparison experiment on “with or without washing” in gel


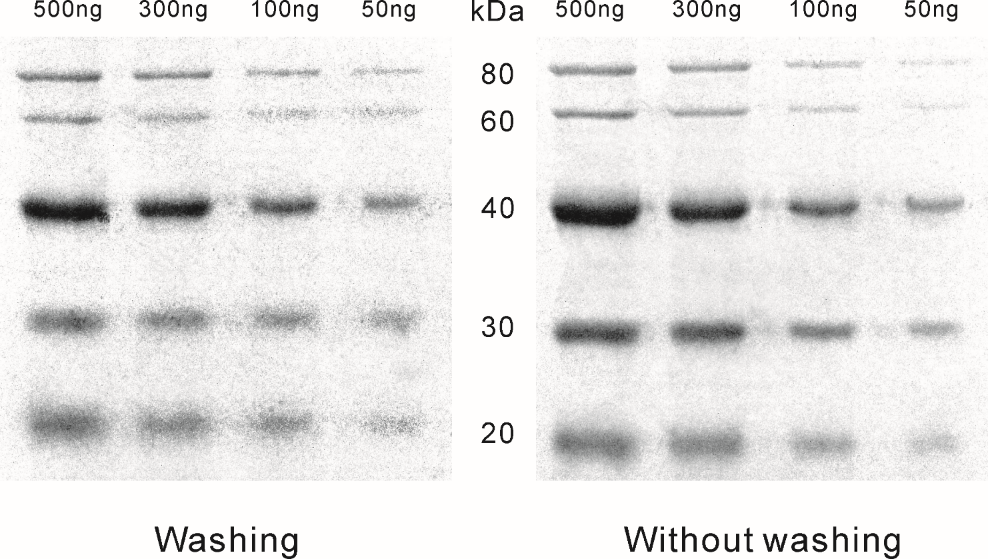


**Figure S2** Protein staining in SDS-PAGE with PyMDI-Zn. Protein samples were commercial protein markers, which contain proteins of 80 kDa (100ng/μl), 60 kDa (100ng/μl), 40 kDa (200 ng/μl), 30 kDa (100ng/μl), and 20 kDa (100ng/μl). Different amounts of protein marker were separated by 12% SDS-PAGE. These gels were stained with PyMDI-Zn for 5 minutes with or without washing.

1. Interference experiments in the presence of non-protein substances


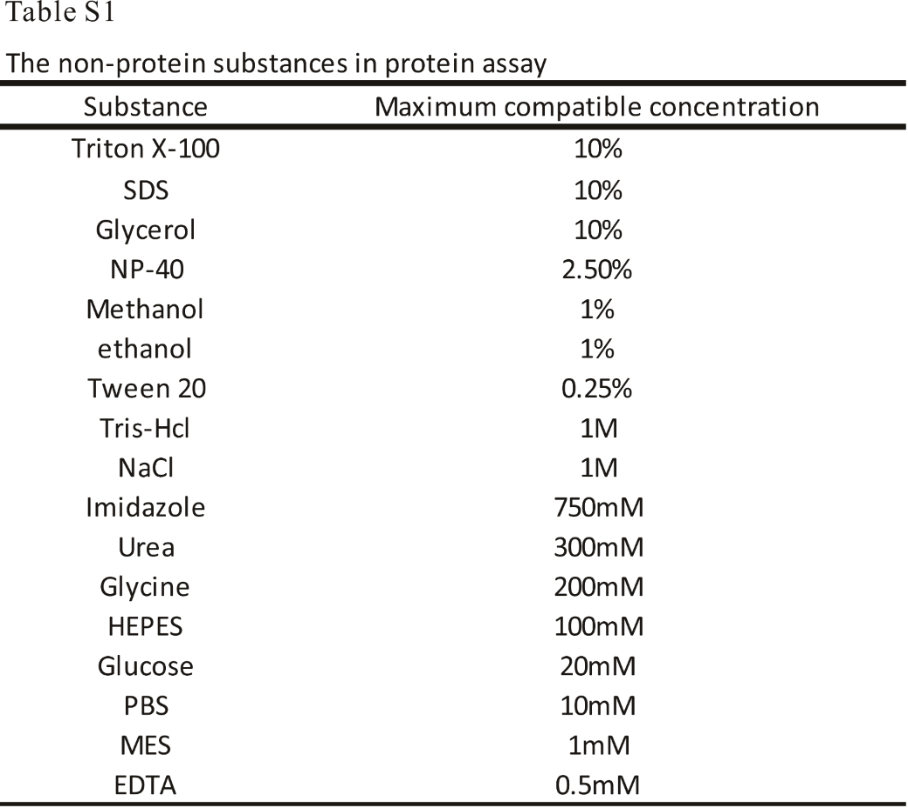


**Table S1** The responses of PyMDI-Zn-BSA complex to various non-protein substances were tested. All the experiments were carried out at a concentration of 100 μM of BSA mixed with 200 μM of PyMDI-Zn in the presence of a different amounts of substances. The fluorescence intensity of PyMDI-Zn-BSA would be relatively stable (not reduced by 10%) even the concentration of corresponding non-protein substance reaches the maximum compatible concentration.


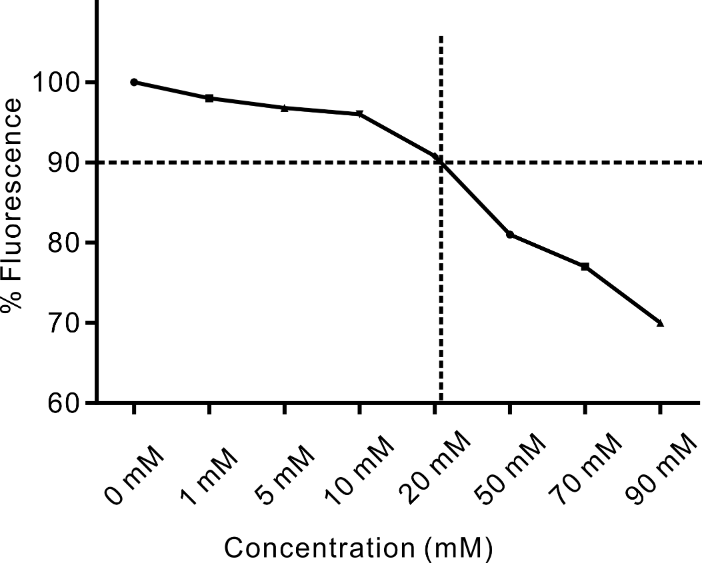


**Figure S3** The responses of protein-PyMDI-Zn to substances (Glucose) were tested to investigate the interference of these substances with the correct protein estimation. All the tests were carried out at a concentration of 100 μM of BSA mixed with 200 μM PyMDI-Zn in presence of a different concentration of foreign substances (Glucose). The maximum concentrations of Glucose which give the perturbation of fluorescence intensity less than 10% can be obtained, the fluorescence of BSA-PyMDI-Zn mixture as a positive control.

1. Comparison experiment on SYPRO Orange and PyMDI-Zn


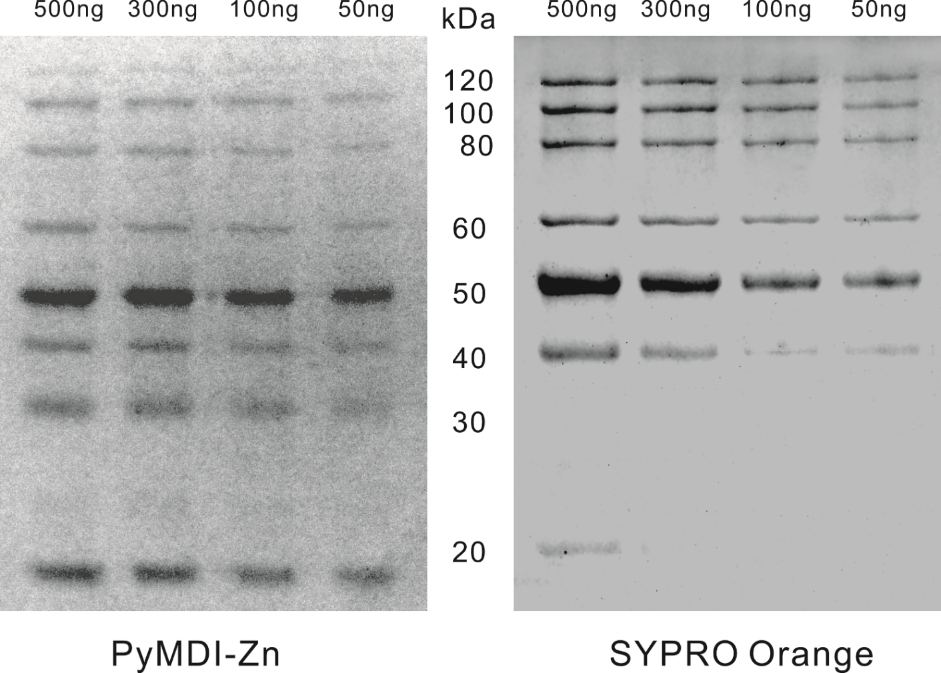


**Figure S4** Protein staining in SDS-PAGE with PyMDI-Zn and SYPRO Orange. Protein samples were commercial protein markers, which contain proteins of 120 kDa (100ng/μl), 100 kDa (100ng/μl), 80 kDa (100ng/μl), 60 kDa (100ng/μl), 50 kDa (200 ng/μl), 40 kDa (100ng/μl), 30 kDa (100ng/μl), and 20 kDa (100ng/μl). Different amounts of protein marker were separated by 12% SDS-PAGE. These gels were stained with PyMDI-Zn for 5 minutes without washing, stained with SYPRO Orange for 30 min.

1. Cellular location with PyMDI-Zn


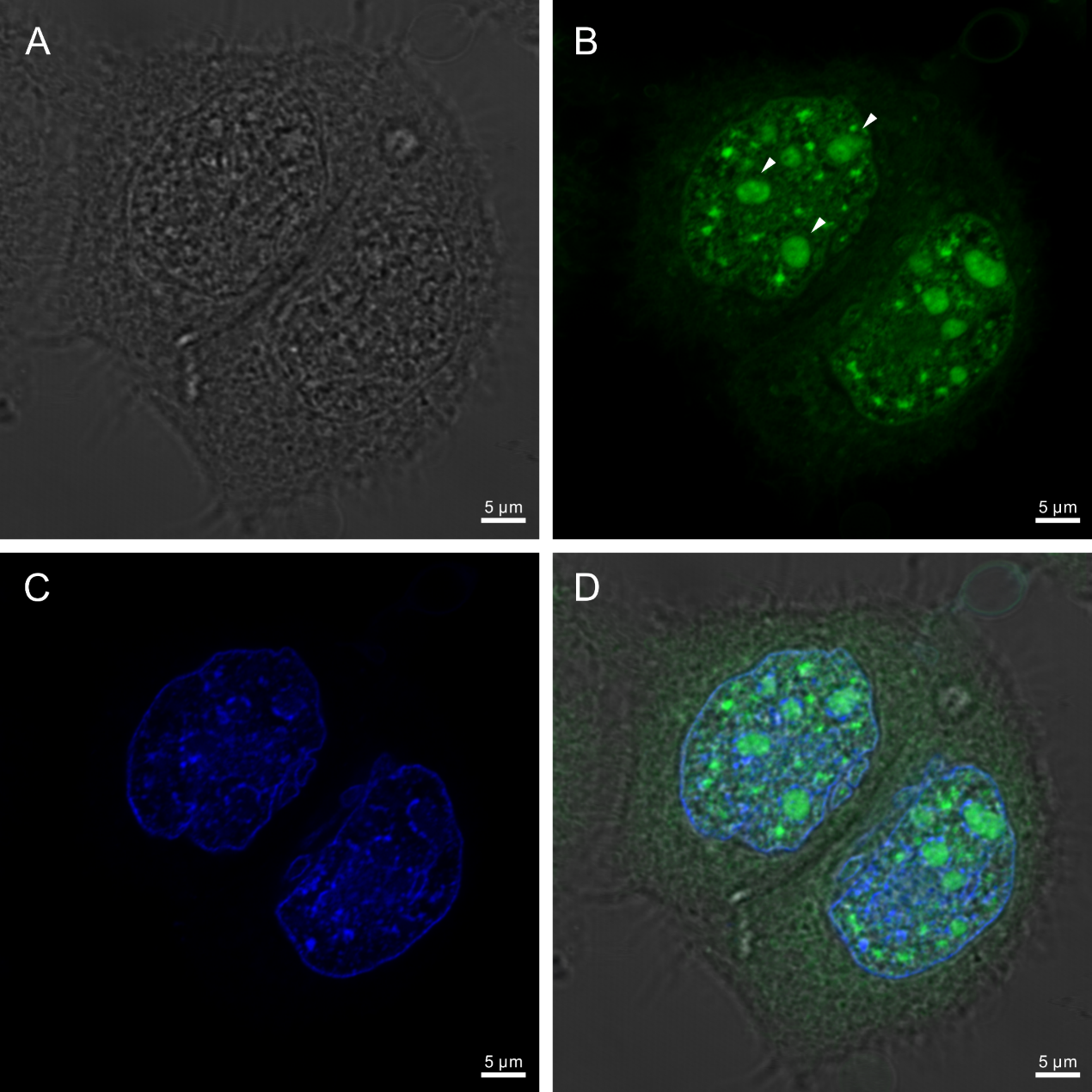


**Figure S5** Hela cells were counterstained with PyMDI-Zn and DAPI. Hela cells grown in 20 mm glass bottom cell culture dish, cells were washed with PBS for 3 times, followed by incubation with PyMDI-Zn (50 μM) and DAPI (nucleus location) for 30 minutes at 37 ℃. (A) bright-field image. (B) Hela cells were stained with PyMDI-Zn (Ex/Em: 488/520 nm). Cells were basically green-stained, and the nucleus areas show higher brightness under laser irradiation. (C) Hela cells were stained with DAPI (Ex/Em: 405/450 nm). (D) the merged image of PyMDI-Zn and DAPI. Scale bars 5 μm


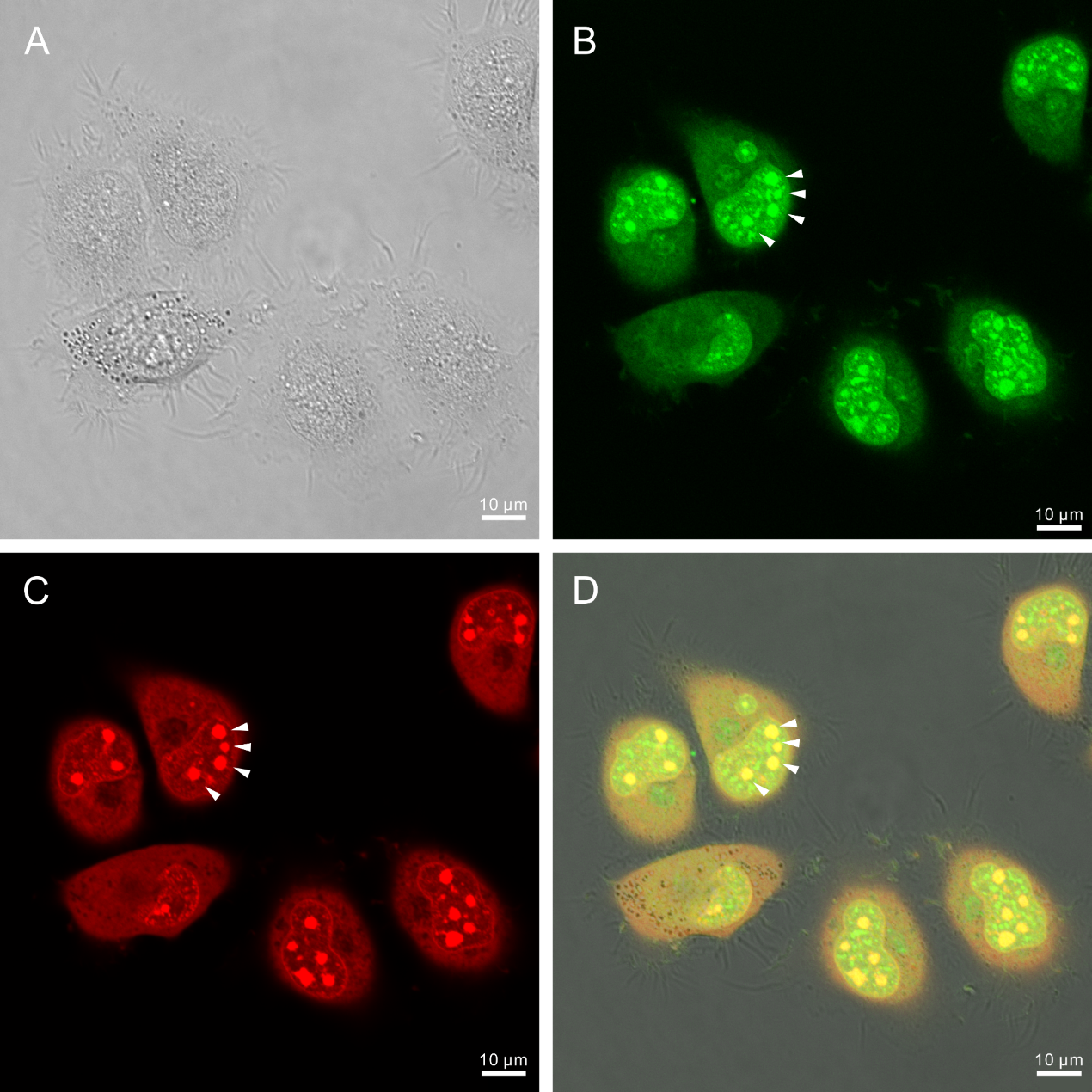


**Figure S6** Hela cells were counterstained with PyMDI-Zn and DAPI. Hela cells grown in 20 mm glass bottom cell culture dish, cells were washed with PBS for 3 times, followed by incubation with PyMDI-Zn (50 μM), DAPI and Pyronin Y for 30 minutes at 37 ℃. (A) bright-field image (B) Hela cells were stained with PyMDI-Zn (Ex/Em: 488/520 nm). There were some highlight particles that appeared in nucleus (white arrowhead). (C) Hela cells were stained with Pyronin Y (Ex/Em: 552/580 nm). Pyronin Y, a nucleolus probe, indicated that those highlight particles were the nucleolus (white arrowhead). (D) the merged image of PyMDI-Zn, and Pyronin Y. Scale bars 10 μm

1. Cell viability assays


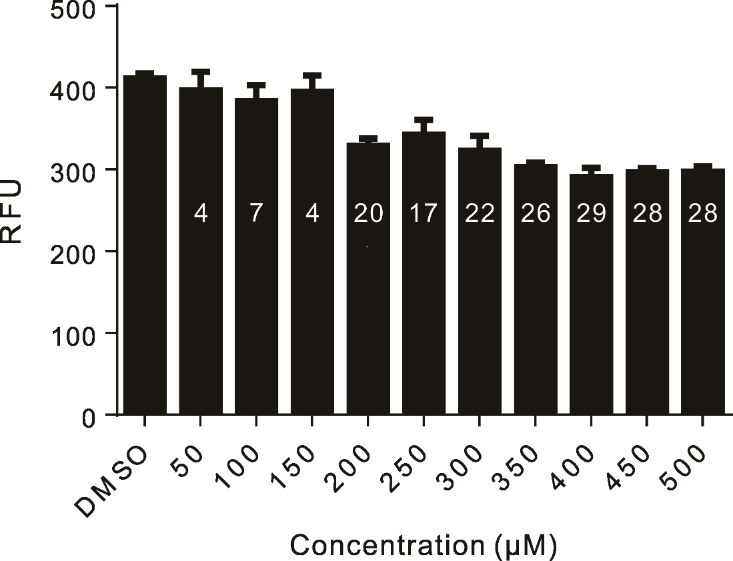


**Figure S7** Cells cytotoxicity was measured using the Alamar blue assay. HepG2 was cultured in a 96-well plate, the cells were exposed to PyMDI-Zn at a concentration in the range of 50~500 μМ (DMSO as a control) for 24 hours, then cytotoxicity was measured using the Alamar blue assay. The excitation wavelength was 530 nm; emission was 580 nm. The white values in the black column represent the percentage decrease. This result showed that PyMDI-Zn would not reduce the cell viability of HepG2 cells with concentrations of less than 200 μМ.

1. Protein quantitation


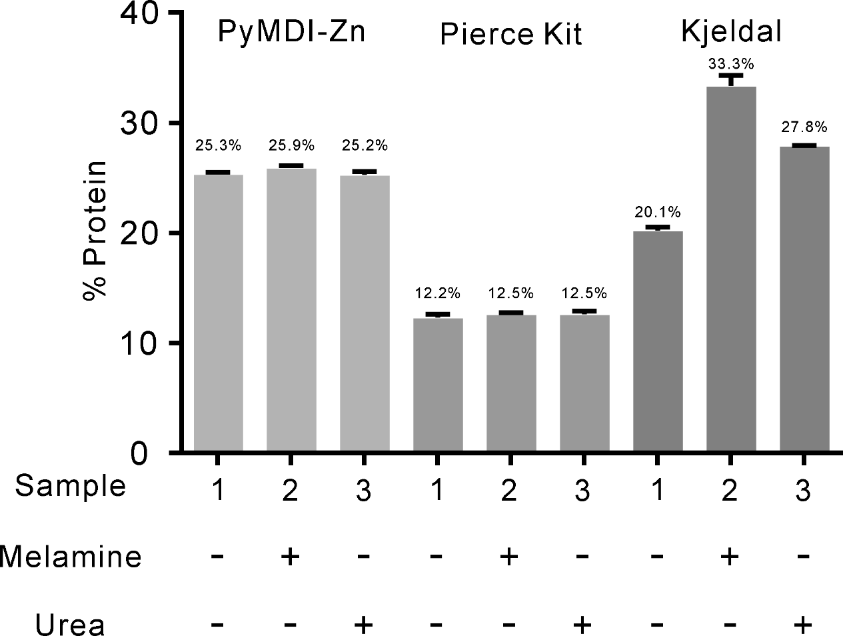


**Figure S8** The determination of total protein using the Kjeldahl method is commonly and officially recognized as a standard method. The Thermo Scientific Pierce Rapid Gold Protein Assay Kit is a rapid protein assay. Sample 1: milk powder (labeled with a protein concentration of 18% by producer); Sample 2: milk powder with melamine to make the protein concentration up to 35% (calculation by nitrogen %); Sample 3: milk power with urea to make the protein concentration up to 30% (calculation by nitrogen %). Samples were tested by PyMDI-Zn, Pierce Kit and Kjeldahl.

1. Notes and references

1. A. Baldridge, K. M. Solntsev, C. Song, T. Tanioka, J. Kowalik, K. Hardcastle and L. M. Tolbert, *Chemical Communications*, 2010, **46**, 5686-5688.
